# Electrospun DegraPol Tube Delivering Stem Cell/Tenocyte Co-Culture-Derived Secretome to Transected Rabbit Achilles Tendon—In Vitro and In Vivo Evaluation

**DOI:** 10.3390/ijms26125457

**Published:** 2025-06-06

**Authors:** Julia Rieber, Iris Miescher, Petra Wolint, Gabriella Meier Bürgisser, Jeroen Grigioni, Jess G. Snedeker, Viola Vogel, Pietro Giovanoli, Maurizio Calcagni, Johanna Buschmann

**Affiliations:** 1 Department of Plastic Surgery and Hand Surgery, University Hospital Zurich, 8091 Zurich, Switzerland; julia.rieber@usz.ch (J.R.); iris.miescher@usz.ch (I.M.); petra.wolint@usz.ch (P.W.); gabriella.meierbuergisser@usz.ch (G.M.B.); pietro.giovanoli@usz.ch (P.G.); maurizio.calcagni@usz.ch (M.C.); 2 Institute for Biomechanics, ETH Zurich, 8092 Zurich, Switzerland; jeroen.grigioni@balgrist.ch (J.G.); jess.snedeker@hest.ethz.ch (J.G.S.); 3 Institute for Biomechanics, Balgrist University Hospital, University of Zurich, 8008 Zurich, Switzerland; 4 Laboratory of Applied Mechanobiology, Department of Health Sciences and Technology, ETH Zurich, 8092 Zurich, Switzerland; viola.vogel@hest.ethz.ch

**Keywords:** mesenchymal stem cells, tenocytes, secretome, rabbit, tendon healing, adhesion, biomechanics

## Abstract

Tendon ruptures have recently reached incidences of 18–35 cases/100,000 and often lead to adhesion formation during healing. Furthermore, scar formation may result in inferior biomechanics and often leads to re-ruptures. To address these problems, we cultivated rabbit adipose-derived stem cells in a co-culture with rabbit Achilles tenocytes and harvested their secretome. Following a cell-free approach, we incorporated such secretome into an electrospun tube via emulsion electrospinning. These novel implants were characterized by SEM, the WCA, and FTIR. Then, they were implanted in the rabbit Achilles tendon full transection model with an additional injection of secretome, and the adhesion extent as well as the biomechanics of extracted tendons were assessed three weeks postoperatively. The fiber thickness was around 3–5 μm, the pore size 11–13 μm, and the tube wall thickness approximately 265 μm. The WCA indicated slightly hydrophilic surfaces in the secretome-containing layer, with values of 80–90°. In vivo experiments revealed a significant reduction in adhesion formation (−22%) when secretome-treated tendons were compared to DegraPol^®^ (DP) tube-treated tendons (no secretome). Furthermore, the cross-sectional area was significantly smaller in secretome-treated tendons compared to DP tube-treated ones (−32%). The peak load and stiffness of secretome-treated tendons were not significantly different from native tendons, while tendons treated with pure DP tubes exhibited significantly lower values. We concluded that secretome treatment supports tendon healing, with anti-adhesion effects and improved biomechanics at 3 weeks, making this approach interesting for clinical application.

## 1. Introduction

Tendon ruptures occur with an incidence of approximately 18–35 cases per 100,000 in the patient population [1,2] and require biophysical stimulation and/or surgery for recovery. The slow and tedious healing of ruptured tendons often goes along with scar formation, leading to a mechanically inferior tissue prone to re-rupture [3]. Furthermore, there is a pronounced tendency for fibrotic adhesions of the healing tendon to the surrounding tissue, resulting in a reduced range of motion.

The main phases of tendon healing include an initial short inflammatory phase, where red blood cells, platelets, and neutrophils enter the rupture site, accompanied by hematoma formation. Typically, growth factors and cytokines that attract further cells are released [4], massively increasing the local cell density in the second proliferative phase and resulting in debris removal and deposition of the new extracellular matrix (ECM). Finally, in the consolidation stage, the fibers are re-arranged from random to parallel in the direction of the force transmission [4,5].

In the last few years, several strategies have been utilized that include bioactive agents to support tendon healing; Transforming Growth Factor beta (TGF-β) delivery to the wound site to accelerate collagen expression and in turn enhance biomechanics [6], Interleukin-1 receptor antagonist (IL1RA) and Platelet Derived Growth Factor-BB (PDGF-BB) supplementation to increase cell proliferation [7], Vascular Endothelial Growth Factor (VEGF) supplementation for a pro-angiogenic stimulation [8], or the application of platelet-rich plasma [9], rich in essential factors for wound healing towards a more regenerative outcome and to prevent adhesion formation. Moreover, cell-based approaches have been reported that targeted tendon injuries by applying tendon-derived stem cells (TDSCs), highlighting the essential roles of mechanical and molecular stimuli in TDSCs for in vitro differentiation [10]. Further cell-based strategies are based on adipose-derived stem cells (ASCs) [11], secretome harvested from ASCs grown on substrates with different topography [12], or ASCs-derived exosomes [13]. In addition, a growth factor-stimulated TDSC-conditioned medium was reported to successfully promote tendon healing, resulting in the enhanced viability and migration capacity of tenocytes, the better alignment of collagen fibers, improved ECM protein expression, and a substantially enhanced biomechanical outcome in rat Achilles tendons [14].

**Figure 1:**
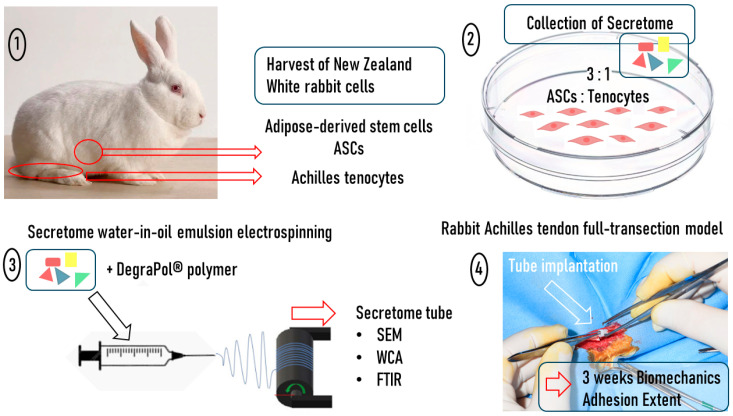
An overview of the experiments conducted in this study. First, adipose-derived stem cells and Achilles tenocytes were harvested from New Zealand White rabbits (**1**). Second, a co-culture of rabbit ASCs and rabbit tenocytes in a ratio of 3:1 was used to collect the secretome (indicated by a scheme of colored triangles and rectangles) (**2**). Third, after concentrating this secretome, a water-in-oil emulsion was made where the secretome was in the aqueous phase and the polymer DegraPol^®^ was in the chloroform/HFP phase. This emulsion was electrospun to yield a bilayer tubular implant material that was characterized by SEM, the WCA, and FTIR (**3**). Fourth, the tube was implanted in a rabbit full-transection model. Upon harvest, the biomechanics of the Achilles tendons and their adhesion extent were assessed three weeks post-operation (**4**).

As co-cultures have been shown to be superior in the support of tendon healing, exemplified by the comparison of ASCs with an ASC/tenocyte co-culture in vitro, where the co-culture significantly increased proliferation and stem cell differentiation [15], we decided to harvest adipose-derived stem cells and Achilles tenocytes from New Zealand White rabbits, grow them in a 3:1 ratio, and collect their secretome (Figure 1). The secretome that was previously characterized in vitro and in a set of biological assays, where particularly tendon-associated proteins, such as tenascin-C, were upregulated in the mixed secretome compared to the pure ASC-derived secretome [16], was concentrated and incorporated in an electrospun DegraPol^®^ tube, following protocols established earlier [17,18,19]. After an in-depth characterization of this secretome tubular implant by scanning electron microscopy (SEM), the water contact angle (WCA), and Fourier-Transformed Infrared Spectroscopy (FTIR), the secretome tubes were implanted in a rabbit full transection Achilles tendon model with an additional initial injection of 50 μL of secretome [20], where 3 weeks post-operation, the biomechanical properties and the adhesion formation were determined and compared to the vehicle without secretome.

The hypothesis of this study was that a tubular implant placed tightly around a sutured tendon and releasing secretome to the healing wound site with an additional initial injection of 50 μL of secretome would enhance biomechanics and reduce adhesion formation in the rabbit full-transection Achilles tendon model compared to a tube without secretome.

## 2. Results

### 2.1. Electrospun Mesh Characterization

**Figure 2:**
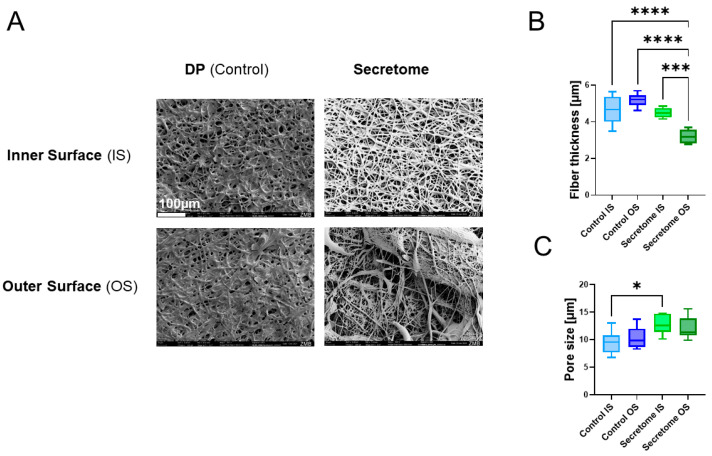
SEM images of the inner and outer surface of electrospun pure DegraPol fiber meshes (DP = control) or secretome/DP meshes (Secretome); (**A**) fiber thickness (**B**) and pore size (**C**). Data is shown as box-and-whisker plots, showing the interquartile range and the full data range. The normality was assessed using the Shapiro–Wilk test. As data were normally distributed, group comparisons were performed using a one-way ANOVA followed by Tukey’s multiple comparisons test (DP, n = 9, secretome, n = 6). *p*-values ≤ 0.05 were considered significant and are denoted as follows: * *p* ≤ 0.05; *** *p* ≤ 0.001, and **** *p* ≤ 0.0001.

The emulsion electrospun secretome-containing scaffolds were imaged with SEM on the inner (IS) and the outer surface (OS) of the tubular implant material (Figure 2). The fiber thickness was significantly smaller for the outer surface of the secretome tubes (3.2 µm ± 0.4) compared to the inner surface (4.5 µm ± 0.3) and compared to the inner (4.6 µm ± 0.7 and outer surface (5.2 µm ± 0.3) of the pure DP meshes that served as a control. As for the pore size, it was significantly larger for secretome tubes on the inner (12.7 µm ± 1.8) surface compared to the inner surface of pure DP meshes (9.5 µm ± 2.0). The tube wall thickness was 265 ± 23 μm. The tubes were 1 cm long and had a diameter of 3 mm.

### 2.2. Water Contact Angles

**Figure 3:**
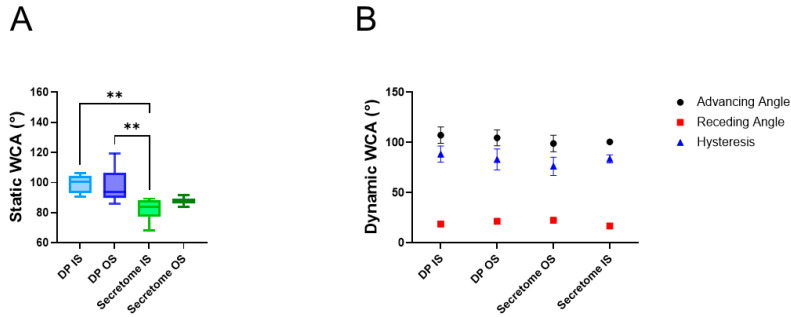
Static (**A**) and dynamic water contact angles (**B**) of pure DP meshes (=control) and of emulsion electrospun secretome/DP fiber meshes. Data is shown as box-and-whisker plots, showing the interquartile range and the full data range. The normality was assessed using the Shapiro–Wilk test. As data were normally distributed, group comparisons were performed using a one-way ANOVA followed by Tukey’s multiple comparisons test. For DP, n = 9; for secretome, n = 6 (**A**). Data is shown as the mean and SD. The Kruskal–Wallis test with Dunn’s multiple comparison was used for comparisons of advancing, receding angle, and hysteresis (**B**). *p*-values ≤ 0.05 were considered significant and are denoted as ** *p* ≤ 0.01.

The assessment of the static WCA demonstrated that the inner surface of the secretome/DP mesh was significantly more hydrophilic (82.3° ± 7.7°) than both surfaces of the DP mesh (Figure 3), indicated by the lower static WCA. The dynamic WCA measurements revealed no statistically significant differences between the four groups, nor for the advancing and the receding angles. Moreover, there was a large hysteresis (76.4°–88.5°) for all groups—also not significantly different among the groups—indicating high heterogeneity for the inner and outer surfaces of both fiber meshes.

### 2.3. FTIR Spectra

**Figure 4:**
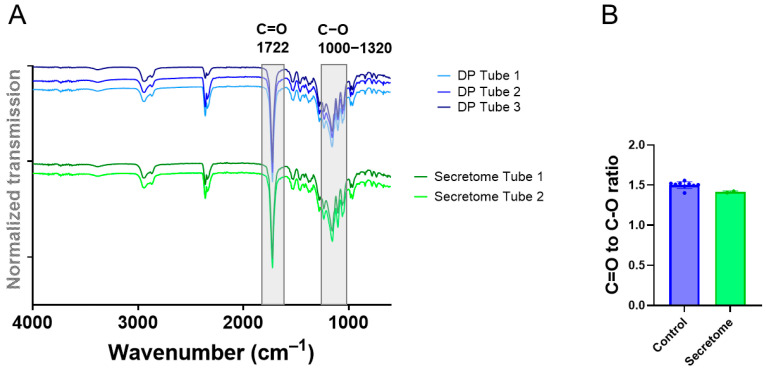
FTIR spectra of three pure DP tubes (Control) and two secretome/DP tubes (Secretome) (**A**) and the calculated C=O-to-C–O ratio of intensities for the two materials (**B**). Data is shown as the mean and SD. The Mann–Whitney test was used for comparisons of the C=O-to-C–O ratio (**B**). No significant difference could be determined.

The pure DP and the secretome/DP meshes were characterized by FTIR (Figure 4). The spectra looked very similar for both kinds of materials. The C=O-to-C–O peak intensity ratio was not significantly different.

### 2.4. Tube Implantation and Extraction

**Figure 5:**
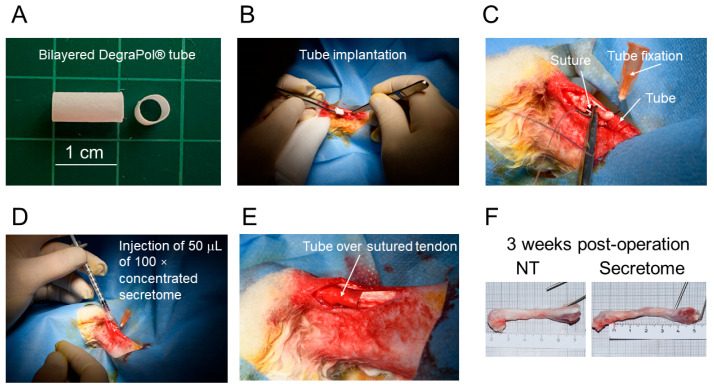
The tube before implantation (**A**). After flipping the tube inside-out to provide the secretome layer towards the tendon, it was pulled over the proximal stump of the fully transected Achilles tendon (**B**). To avoid back slippage, the tube was fixed in position by a cannula during suturing (**C**). A volume of 50 μL of 100× concentrated secretome was injected after finishing the suture (**D**). After the removal of the cannula, the tube was pulled over the sutured tendon (**E**). Three weeks post-operation, the extracted tendons of secretome-treated and contralateral non-treated (NT) tendons appeared similar during macroscopic inspection (**F**).

The tubes were 1 cm long with a wall thickness of 265 ± 23 μm, and were flipped and rolled up before implantation (Figure 5). During suturing, the tubes were fixed in position by the insertion of a cannula. Three weeks post-operation, the secretome-treated tendons had a similar macroscopic appearance to the contralateral non-treated tendons.

### 2.5. Adhesion Formation in the Rabbit Achilles Tendon Full Transection Model

**Figure 6:**
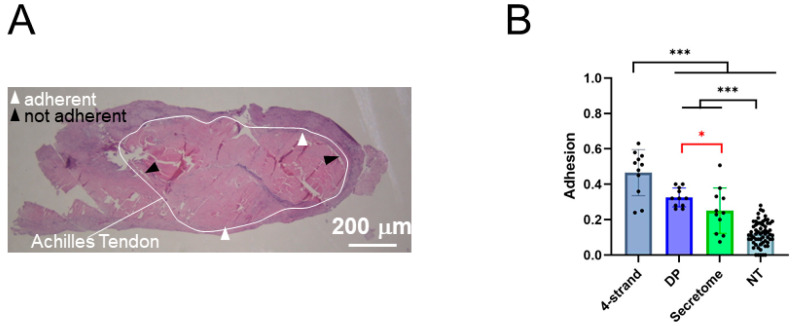
From histological cross-sections, the adhesion extent was assessed as a fraction by measuring the adherent distances and dividing the sum of them by the whole circumference, exemplified with H&E-stained sections (**A**). The fraction of adherent tissue is presented for the four experimental groups; non-treated contralateral legs that did not have any intervention (NT), repaired tendons with a pure DP tube (DP), repaired tendons with a secretome/DP tube (secretome), and repaired tendons without a tube (4-strand, indicating the type of suture used to repair the tendon) (**B**). Data is shown as the mean and SD. The normality was assessed using the Shapiro–Wilk test. As the data were normally distributed, group comparisons were performed using a one-way ANOVA followed by Fisher’s PLSD multiple comparisons test. (n = 11 for 4-strand, DP and secretome, n = 61 for NT [20]). *p*-values ≤ 0.05 were considered significant and are denoted as follows: * *p* ≤ 0.05, *** *p* ≤ 0.001.

Three weeks post-operation, the adhesion extent of the rabbit Achilles tendons to the surrounding tissue was quantified by dividing the sum of all of the adhesions’ length by the whole distance of the circumference (Figure 6). The adhesion formed in a mere 4-stand sutured tendon was significantly higher (0.47 ± 0.13 than all other experimental groups, while the adhesion of the non-treated tendons (healthy collateral tendons) was significantly lower (0.12 ± 0.07) than all other experimental groups. The adhesion fraction of the secretome/DP tube-treated tendons was significantly lower (0.25 ± 0.13) than the adhesion formed by a pure DP tube (0.33 ± 0.05).

### 2.6. Morphological and Biomechanical Properties of Repaired Tendons

**Figure 7:**
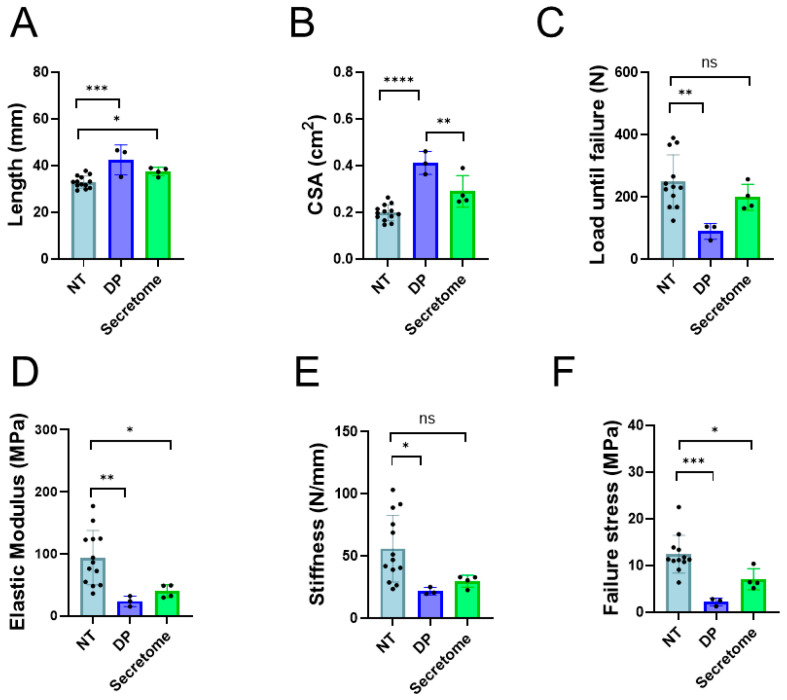
The biomechanical properties of the repaired tendons, either treated with a pure DP tube (DP) or with a secretome tube and a secretome injection (Secretome), compared to healthy non-treated tendons (NT). Data is shown as the mean and SD for length (**A**), cross sectional area (CSA) (**B**), load until failure (**C**), elastic modulus (**D**), stiffness (**E**), and failure stress (**F**). The groups were compared using the nonparametric Kruskal–Wallis test for the length, elastic modulus, and stiffness (no normal distribution of data). As data were normally distributed, groups of the following parameters were compared using a one-way ANOVA: CSA, load until failure, and failure stress. *p*-values ≤ 0.05 were considered significant and are denoted as follows: * *p* ≤ 0.05; ** *p* ≤ 0.01, *** *p* ≤ 0.001, **** *p* ≤ 0.0001; not significant (ns).

After three weeks in vivo, the rabbit Achilles tendons were measured morphologically and biomechanically, and a significant increase in length was determined for DP tube-treated tendons (42.5 mm ± 6.4) as well as for secretome/DP-treated tendons (37.5 mm ± 1.8), although the significant difference was less pronounced in the pair-wise comparison of NT and secretome compared to NT and DP (Figure 7). The cross-sectional area (CSA) was significantly higher for DP tube-treated tendons (0.41 cm^2^ ± 0.05) compared with NT (0.20 cm^2^ ± 0.03) and with secretome-treated tendons (0.29 cm^2^ ± 0.07). Importantly, the secretome-treated tendons had a non-significantly higher CSA than NT.

As for the biomechanics, the load-until-failure was significantly higher in NT tendons (249.5 N ± 86.5) compared to DP tube-treated tendons (90.0 N ± 25.1) but not compared to the secretome group (199.3 N ± 42.2). Moreover, the failure stress was significantly higher for the NT group (12.5 MPa ± 4.0) compared to the two other experimental groups. The stiffness was significantly higher for the NT group (56.0 N/mm ± 26.8) compared to the DP group (21.94 N/mm ± 2.96), however, not compared to the secretome group (29.95 N/mm ± 4.90). The elastic modulus was significantly higher for the NT group (93.8 MPa ± 43.7) compared to both other experimental groups.

## 3. Discussion

Cell-free approaches for tendon rupture repair belong to an increasing field of research because circumventing tedious and time-dependent cell expansion to provide enough cells to the patient at a distinct time point remains attractive for surgeons. Among these approaches, diverse strategies have been proposed, such as exosomes harvested from adipose-derived stem cells [21] or from tendon stem/progenitor cells [22], and a decellularized tendon graft as a matrix combined with BMP-2 delivery [23] or decellularized tendon-derived stem cell sheets [24]. Given that many approaches show promising results in pre-clinical models, the rationale of the current study was to use secretome harvested from a co-culture of rabbit ASCs and rabbit Achilles tenocytes to be incorporated in a drug delivery system and applied in a rabbit full-transection Achilles tendon model, which was hypothesized to improve biomechanics and reduce adhesion formation of the healing tendon.

To this end, we harvested ASCs and tenocytes from New Zealand White rabbits in a previous study and cultivated them either as a pure ASC culture or as a co-culture of ASCs and tenocytes (mixed) in a ratio of 3:1 according to the promising results published by Kraus et al. [15]. While the ASC-derived secretome was composed of 2774 proteins, the mixed secretome had 2693 proteins, from which 182 proteins were significantly different in these two secretomes [16]. Differential analysis revealed that the mixed secretome contained significantly higher amounts of tenascin-C, collagen XI, aggrecan, and TGF-β [16], which are important as structural components and are known as the ECM-related growth factor (TGF-β) involved in tendon healing [3]. Moreover, ligand–receptor analysis revealed the differential upregulation of ligand–receptor pairs for the mixed secretome but not for the secretome obtained from only ASC culture. Specifically, significantly enriched signaling pathways included the Ras, Rap1, PI3K Akt, MAPK, and HIF-1 pathways, all important during tendon healing, as they affect proliferation, differentiation, cell growth, survival, inflammation, metabolism, and angiogenesis, respectively [16]. In summary, the comparison of these two secretomes indicated that the differential expression of matrix-related proteins in the co-culture-derived secretome was more promising for tendon repair than the one derived from the ASC culture [16]. Hence, we used this co-culture-derived secretome for further in vivo investigation presented here.

First, we incorporated the secretome into a DegraPol^®^ tube via water-in-oil emulsion electrospinning, following the same approach that was utilized to incorporate PDGF-BB [17], IGF-1 [18], or TIMP-1 [19], respectively. Then, we characterized this novel implant material through the assessment of fiber thickness, pore size, the water contact angle, and FTIR. Finally, we implanted the secretome tube into the rabbit Achilles tendon full transection model and analyzed the biomechanics of the extracted tendons three weeks post-operation. In addition, the adhesion extent to the surrounding tissue was examined.

As a first characterization step for our novel electrospun secretome tube, we assessed the fiber thickness via SEM. While pure DP fibers (control) exhibited fiber diameters between 4 and 5 μm and were not significantly different on the inner and outer layer of the mesh, the fiber thickness of the secretome-containing fibers were smaller, particularly on the outer surface where they were approximately 3 μm thick, which was significantly smaller than on the inner surface and compared to the DP fibers (Figure 2B). The comparison of these data to other bioactive electrospun meshes, such as those designed for the delivery of ibuprofen to the surrounding tissue in a 2-layer mat with an average fiber diameter of 2 μm [25], reveals similar thickness. However, other drug-releasing electrospun meshes have been reported to exhibit smaller fibers, like Exendin-4 (a peptide growth factor) releasing mats with a fiber thickness of 0.6–1.0 μm [26] or a basic Fibroblast Growth Factor (bFGF)-releasing mesh with 0.1 μm fiber thickness [27]. Nevertheless, for DP-based emulsion electrospun fibers, we measured a very similar fiber thickness as found for the secretome tube, and can confirm that the emulsion electrospun fibers tend to have smaller thicknesses than pure DP fibers have [18], as long as the weight percentage of dissolved DP in the chloroform/HFP solution utilized for electrospinning is lower than 12 wt % [17].

According to the lower fiber thickness found in secretome emulsion electrospun fibers compared to pure DP fibers, the pore size showed opposite trends, with larger pores found for secretome fiber meshes and with significantly larger pores on the inner side of the mesh (Figure 2C). The pore size was in the range of 10–14 μm, facilitating the easy cell invasion of tenocytes into the pores. Although tenocytes have a size of 8–10 μm in their round form, they are reported to reach sizes up to 50 μm when elongated [28,29], indicating that the penetration of the porous tubular implant material would rather be hindered and, in turn, diminish adhesion formation.

The water contact angles of electrospun meshes highly depend on the polymer used. Examples of very hydrophilic meshes have been reported for poly vinyl alcohol/chitosan meshes that were cross-linked with glutaraldehyde and exhibited WCAs in the range of 28–37° depending on the drugs that were incorporated, such as gallic acid, which led to even more pronounced hydrophilicity due to an increase in hydroxyl groups [30]. On the other end of the WCA scale are very hydrophobic materials like fluorinated poly(aryl ether)/polypropylene composite meshes with a WCA of 151° [31]. Moderately hydrophobic poly(lactide-co-ε-caprolactone) has been reported to have a WCA of 105° [32], which is similar to our findings for the inner and outer surfaces of the pure DP fiber mesh (Figure 3A). As expected, the incorporation of an aqueous secretome solution into the DP polymer by emulsion electrospinning lowered the WCA significantly, at least on the inner surface of the secretome mesh, where we determined approximately 89°, indicating a slight hydrophilicity, and confirming previous results found for IGF-1 protein incorporation into DP fibers by emulsion electrospinning [18]. In addition, the dynamic WCAs were very similar, as reported earlier [18] for pure DP fibers and for secretome emulsion electrospun DP fibers (Figure 3B). The high hysteresis found for both kinds of materials indicates the heterogeneous surfaces, which was expected from the SEM images taken of these surfaces, where the fibrous structure and obvious heterogeneity could be verified (Figure 2A).

The FTIR spectra assessed for pure DP meshes and for secretome meshes did not show any obvious differences, with similar C=O-to-C–O intensity ratios (Figure 4) confirming our expectations because the amount of proteins incorporated via water-in-oil emulsion electrospinning was relatively small compared to the amount of polymer. Other electrospun drug delivery systems, however, report differential FTIR spectra for drug-loaded delivery systems compared to vehicles. For instance, a core-shell fiber mesh with a curcumin/hydroxypropyl-β-cyclodextrin inclusion complex as the core and pullulan as the shell exhibits a different FTIR spectrum than pullulan alone [33].

In order to elucidate the in vivo performance of the secretome, we applied it in the previously established rabbit full transection Achilles tendon model [20]. The secretome tube was pulled over the sutured tendon after the injection of 50 μL of the concentrated secretome between the tendon stumps, enabling an in situ initial boost of 225 μg proteins which were supposed to additionally support the tendon healing. We used this amount of proteins because Oryan and Moshiri reported that a concentration of 1.2 μg/kg_rabbit_ of bFGF applied on days 3, 7, and 10 post-injury in the New Zealand White rabbit model led to higher cell density, thicker collagen fibers, improved biomechanics, and also lower adhesion formation [34]. With 225 μg applied once to a 4 kg rabbit, we had a concentration of 55 μg/kg_rabbit_ of secretome, which was higher than values by Oryan and Moshiri reported to be on the safe side and for sure to evoke an in vivo response. Three weeks post-operation, we assessed the adhesion formation of the Achilles tendon to the surrounding tissue by a method established by Tan et al. [35]. The secretome-treated tendons had a significantly lower adhesion formation compared to a sutured tendon that received a pure DP tube without secretome injection or secretome incorporated in the tube (Figure 6). Multiple factors contribute to postoperative adhesion formation, and inflammation is reported to promote fibrotic adhesions. In this regard, the secretome of adipose-derived stem cells has been reported to mitigate inflammation and, in turn, support wound healing and reduce fibrosis [36,37]. Moreover, the secretome derived from a 3:1 ASC-to-tenocyte co-culture supplemented to a rabbit Achilles tenocyte culture previously showed an induction of ALOX 15 gene expression, indicating the pro-resolving impact of this specific secretome [16]. In addition, this secretome induced the downregulation of α-SMA in rabbit Achilles tenocytes, which also points towards an anti-fibrotic effect, as α-SMA overexpression is associated with a trans differentiation of tenocytes into myofibroblasts, although the majority of α-SMA-positive myofibroblasts have been reported not to be derived from tenocytes [38,39]. Taken together, the secretome therapeutic approach, here conducted as an initial injection immediately after a conventional tendon suture and a tubular implant with incorporated secretome to be slowly biodegraded and to release secretome over time, significantly reduced adhesion formation at 3 weeks post-operation.

Tendon elongation after Achilles tendon rupture is a common clinical problem, regardless of the initial surgical or conservative approach [2]. The consequences of elongated Achilles tendons are functional deficits, such as reduced plantarflexion strength or a lowered heel-rise maximum height [40]. In order to re-establish the original Achilles tendon length, a case report showed that endoscopic shortening was successful; after cutting the (too long) Achilles tendon, it was re-sutured with shortened stumps in a minimally invasive surgery [41]. Nevertheless, novel approaches that support tendon healing to reach insignificant lengthening or even the full restoration of the initial length are being investigated. For example, Majewski and co-workers have applied the growth factors bFGF, BMP-12 (GDF-7 [42]), and TGFβ1, either applied (i) all together as an initial injection during surgery of a transected rat Achilles tendon, (ii) as tiered injections after surgery, or (iii) by being loaded onto a collagen sponge implanted during surgery, releasing the GFs over time [43]. These authors found that the GF-loaded sponge-treated rat Achilles tendons were as long as the native untreated tendons already at 1 week post-op, while all other treatments resulted in longer tendons [43]. At 8 weeks, however, all other treatment groups also reached the GF-loaded sponge group with regard to length.

In our secretome-treated rabbit Achilles tendon, we found an increase in length of the tendons compared to the native tendon length in New Zealand White rabbits (Figure 7A). However, while the pure DP tube-treated tendons were significantly longer than the native tendons with a *p* < 0.001, the elongation of the secretome-treated tendons was less pronounced—they were significantly longer only with a *p* < 0.05 compared to native tendons, indicating an improvement towards shorter tendons during healing.

In addition to the length of the healing Achilles tendon, the CSA is also an important morphological aspect because healing tendons tend to swell due to increased water uptake caused by upregulated proteoglycans, which should, however, go back to normal levels at the end of the remodeling phase [44]. Furthermore, an enlarged CSA may indicate scar formation characterized by the excessive accumulation of ECM in the healing tendon [45], a problem encountered particularly when a large CSA persists 3 months post-injury [46].

Therefore, a welcome finding in our secretome-treated Achilles tendons was the significantly lower CSA compared to the DP tube-treated tendons (no secretome) (Figure 7B), which may indicate an acceleration in healing (swelling already over) or a lowered fibrotic reaction with less scar formation and a more regenerative way of healing. Furthermore, there was only a trend of a higher CSA for the secretome-treated tendons compared to native tendons, but there was no significant difference. We judge this finding important as it is consistent with the observed lower adhesion formation (Figure 6B) that may also be a result of a reduced fibrotic reaction under secretome therapy.

The load until failure of the extracted tendons showed higher values in the secretome treatment group than for the pure DP tubes, which was, however, only a trend. Nevertheless, although DP-treated tendons had significantly lower peak loads than native tendons, there was no significant difference between native tendons and secretome-treated tendons (Figure 7C). Korcari and co-workers have assessed the biomechanics of transected C57Bl/6J mice flexor digitorum profundus and found that 3 weeks post-surgery scar tissues and tendon/scar composite tissues had similar peak loads, with a trend towards higher peak loads for scars [45]. Furthermore, they reported a similar stiffness, a similar peak stress, and a significantly lower elastic modulus for scar tissue compared with the composite tissue [45]. In our study, there was a higher stiffness in the secretome group compared to DP tubes by trend, but again, as was found for the peak load, a non-significant difference of the secretome group compared with native tendons (Figure 7E). In contrast to the study by Korcari et al., where a significantly lower modulus was determined for pure scar tissue compared with composite tissue, we found no significant difference between the secretome treatment and pure DP tubes for the elastic modulus (Figure 7F).

The trends of higher peak loads and higher stiffness in secretome-treated tendons compared to DP tubes without secretome may, on the one hand, indicate earlier functional recovery and allow the patient to return to daily activities earlier, which has to be judged positively because the risk of a re-rupture even under high loads becomes smaller with these improved biomechanics; they may, on the other hand, be based on scar tissue formation rather than fully regenerated tendon tissue, following Korcari et al.’s results for the interpretation of our findings [45]. As we found a significantly higher total cell density in the core tissue of the tendons treated with secretome, the according higher peak loads may have resulted from more collagen deposition compared to pure DP tube-treated tendons, although the collagen fiber orientation did not show significant differences when compared to DP tube-treated tendons (Supporting Information, Figure S1).

Some limitations of this study have to be mentioned. Although the chosen time point, 3 weeks post-operatively, revealed promising results with respect to adhesion formation and the biomechanical outcome, time points at later stages in tendon healing, particularly at the end of the consolidation stage, may be very interesting to analyze for the same parameters. Furthermore, we tested only one kind of secretome; however, secretome obtained from a pure rabbit ASC culture or a pure rabbit Achilles tenocyte culture would enhance the comparability of the outcome to other conditions if applied in the same pre-clinical model (the rabbit Achilles tendon full transection model).

## 4. Materials and Methods

### 4.1. Synthesis of DegraPol^®^ (DP)

To synthesize DP, a mixture consisting of 25 wt% poly(3-(R-hydroxybutyrate)-co-(ε-caprolactone)-diol; Mn = 2824 g/mol) and 75 wt% poly(ε-caprolactone)-diol-co-glycolide (15 mol% glycolide, 85 mol% ε-caprolactone; Mn = 1000 g/mol) was dissolved in 1,4-dioxane and dried until the water content was reduced to below 20 ppm. The resulting solution was cooled, and a stoichiometric amount of 2,2,4-trimethylhexane-diisocyanate (TMDI) was added. After one day, dibutyltin dilaurate (20 ppm) was incorporated three times within the day to reach a molecular weight range of 100–110 kDa. The polymer was precipitated in cooled hexane isomers, purified using chloroform and a silicagel 60 column (Fluka, Buchs, Switzerland), and further purified through precipitation in cooled ethanol. DP was kindly provided by Ab Medica (Cerro Maggiore, Italy), a batch received in September 2022.

### 4.2. Scaffold Production and Incorporation of Secretome

The polymer solutions were prepared at least one day prior to electrospinning to ensure complete dissolution and stability. For each scaffold, a PEG (35 kDa, Aldrich, Buchs, Switzerland) solution was made by dissolving 1.5 g of PEG in 3.5 g of chloroform (Sigma-Aldrich, Buchs, Switzerland). The DP solution was formulated separately by mixing 0.6 g of DP powder with 3.52 g of chloroform and 0.88 g of 1,1,1,3,3,3-Hexa Fluoro-2-Propanol (HFP, Aldrich, Buchs, Switzerland) in a screw-cap glass container. To incorporate the secretome, produced previously in our laboratory [16], 100 µL of 100× concentrated secretome was added dropwise into the DP solution under continuous stirring at 500 rpm for five minutes using a magnetic stirrer. The resulting mixture was briefly vortexed and subsequently emulsified in an ultrasonic bath for 15 min to ensure proper dispersion. The final emulsion was transferred into a 5 mL glass syringe (Huberlab) and used immediately for electrospinning.

The tubular scaffolds were produced using a custom-designed electrospinning setup that included a DC high-voltage power supply (Glassman High Voltage Inc., High Bridge, NJ, USA), a syringe pump (SP210cZ, WPI, Friedberg, Germany), and a needle holder mounted on a lateral transporter (Euro Star B, IKA Labortechnik, Staufen, Germany). The polymer solution was conveyed through a Teflon hose into a blunt-ended stainless steel needle (1 mm inner diameter, 0.3 mm wall thickness; Angst & Pfister AG, Zürich, Switzerland). This needle dispensed the solution onto 550 mm-long metal rods, which were attached to a rotating motor (Euro Star B, IKA Labortechnik) acting as the fiber collector. Electrospinning was performed at room temperature (22–23 °C) and under controlled humidity conditions (25–35%). The parameters for electrospinning included a flow rate of 1 mL/h, a 19.5 cm distance between the needle tip and the collector, and an applied voltage of 12.5 kV. To ensure uniform fiber deposition, the needle was programmed to move laterally across a 20 cm range, while the collector rotated at 500 rpm. To enable easy scaffold removal, a preliminary layer of PEG was electrospun onto the surface of the metal rod. Once the base layer was established, additional layers consisting of either DP alone or DP blended with secretome were deposited. For scaffold retrieval, 50% ethanol was pipetted directly onto the tubular structures, and the scaffolds were gently released from the collector using tweezers. Following detachment, they were immersed in 50% ethanol, rinsed thoroughly with distilled water, and dried in a desiccator. The drying period ranged from three to seven days, after which the scaffolds were stored at 4 °C until use.

### 4.3. SEM Fiber and Pore Size Analysis

Small sections from each scaffold tube, including both inner and outer surfaces, were prepared for imaging. Three tubes for DP and two for secretome were measured, where for each tube, the fiber thickness and pore size were assessed and were averaged for three individual pictures, resulting in n = 9 for DP and n = 6 for secretome. The samples were affixed to SEM stubs (SEM-Alu-12-6, TAMS, Schwerzenbach, Switzerland) using conductive double-sided adhesive tape (AD-cloth-tab-12, TAMS, Schwerzenbach, Switzerland). All coating and imaging procedures were conducted using equipment maintained by the Center for Microscopy and Image Analysis at the University of Zurich. Prior to imaging, the samples were coated with a 10 nm layer of platinum using a Safematic CCU-010 sputter coater (safematic GmbH, Zizers, Switzerland). SEM was then performed with a Zeiss Gemini SEM 450 (Munich, Germany), operating at an accelerating voltage of 5 kV. Images were captured at 500× magnification using the secondary electron detector, with a brightness setting of 49%. Fiber diameters and tube wall thicknesses were quantified using ImageJ software (version 1.53e/Java 1.8.0_172, 64 bit). Measurements were based on the scale bars provided in the SEM images. For analysis, a diagonal reference line was drawn across each image, and all fibers or pores intersecting this line were measured.

### 4.4. WCA Evaluation

The WCA was assessed for both pure DP tubes and DP emulsion tubes containing secretome. Three tubes for DP and two for secretome were measured in triplicate, resulting in n = 9 for DP and n = 6 for secretome. To prepare the samples, each tube was carefully opened lengthwise using a scalpel and mounted flat onto a glass plate using double-sided adhesive tape to secure both the inner and outer surfaces for measurement. A goniometer equipped with an IDS uEye camera was used to capture the contact angle. Milli-Q water droplets (5 µL each) were dispensed onto the sample surface using a 1 mL syringe. For each droplet, the left and right contact angles were recorded, and their average was used to determine the static WCA. A minimum of three measurements were performed for each sample to ensure accuracy.

Dynamic WCA measurements were also conducted using the same goniometer setup. Starting with a 5 µL droplet, water was either added or withdrawn at a controlled rate of 15 µL/min to determine the advancing and receding contact angles, respectively. Measurements were taken at one-second intervals over the course of one minute. WCA hysteresis was calculated as the difference between the advancing and receding angles.

### 4.5. FTIR Analysis

FTIR spectroscopy was carried out using a Varian 640 FTIR spectrometer (ABB, Zurich, Switzerland) equipped with a Golden Gate diamond ATR unit featuring temperature control. Three tubes for DP and two for secretome were measured in triplicate for DP and once for secretome, resulting in n = 9 for DP and n = 2 for secretome. Spectra were collected over a wavenumber range of 600 to 4000 cm⁻^1^, at a resolution of 4 cm⁻^1^. Each spectrum represented an average of 64 individual scans to enhance the signal quality. For comparative evaluation, the intensity ratio of the C = O absorption peak at 1720 cm⁻^1^ to the C – O peak at 1175 cm⁻^1^ was determined. All spectra were normalized relative to the C = O peak at 1720 cm⁻^1^. The analysis included DP powder, unmodified DP tubes, and DP tubes containing different emulsions. PEG was also examined separately to assess possible contamination. To identify characteristic functional groups, the recorded peaks were compared against values listed in an IR reference spectrum table (Merck KGaA, Darmstadt, Germany).

### 4.6. In Vivo Implantation: Rabbit Achilles Tendon Full Transection Model

For the implantation of the secretome tubes, 4 female New Zealand White rabbits, aged 12–16 weeks and specific pathogen free (SPF), were used (Charles River, Research Models and Services, Sulzfeld, Germany). The animals were housed, maintained, and fed as previously described [20], and acclimatized to their environment for 2 weeks before surgery. Ethical approval for the experiments was obtained from the veterinary office of Zurich, Switzerland (reference numbers ZH 080/2021; 33530). The full transection of the Achilles tendon 2 cm above the calcaneus, followed by a 4-strand Becker suture, was carried out as described earlier [20]. The tubes were sterilized with H_2_O_2_ (plasma sterilization) before implantation and flipped over the wound. The bilayered tube was flipped inside-out because the smooth inner surface, consisting of a pure DP layer that was produced as the first layer on the metal rod, should face the surrounding tissue, while the rough surface made of secretome containing DP fibers should face the repaired tendon. Then, 50 μL of 100× concentrated secretome was injected. Afterwards, the wound was closed with a running suture (using a USP 6.0 polypropylene fibre (SAPEN, Shanghai, China)) and a well-padded cast was applied with an angle of 180° at the ankle. The rabbits received a Durogesic Matrix patch after surgery (Janssen-Cilag AG, Zug, Switzerland) with 4.2 mg Fentanyl per patch to provide analgesia for about 72 h with 25 μg/h Fentanyl. The rabbits were euthanized three weeks later in deep anesthesia (100 mg/kg Ketamine and 4 mg/kg Xylazine) with 80 mg/kg Pentobarbital (Esconarkon ad us. vet., Zurich, Switzerland), and the tendons were removed. Surgery was performed on one hind leg while the counter hind leg was not treated (NT) and served as the control. The extracted tendons were immediately frozen and stored at −20 °C in a gauze moistened with 0.9% NaCl solution.

### 4.7. Adhesion Extent

The tendons were thawed overnight at 4 °C and warmed to RT before they were dehydrated and embedded in paraffin according to commonly established protocols. Cross sections of 5 μm in the wound region (perpendicular to the Achilles tendon) were deparaffinized with xylene and rehydrated prior to histological staining with Hematoxylin–Eosin (H&E) according to commonly established procedures. To quantify the extent of adhesion (n = 11 for 4-strand, DP and secretome, n = 61 for NT), H&E-stained sections, as described by Tan et al. (2010) [35], were analyzed at 8× magnification (Leica EZ4D microscope, Zurich, Switzerland), where data for the groups 4-strand DP and some data for the NT group were taken from previous studies for the comparison of the secretome-treated tendon adhesion assessed here [20]. To calculate the percentage of adhesion, the length of the contact region of the tendon with the surrounding tissue and the whole tendon perimeter were determined with the Synedra View software (version 22.0.0.12), before dividing the length of the contact region by the length of the total perimeter. Further histological analysis included the total cell density in the core tendon tissue, which was counted for three fields of view (FOVs) in each of the three sections prepared from each of the four rabbits. The fields of view were 100 μm × 100 μm and the cell density was then computed for the number per mm^2^. The collagen fiber orientation was assessed semi-quantitatively, with score 1 = native tendon with practically no undulated fibers; 2 = slightly undulated fibers; 3 = undulated fibers; 4 = strongly undulated fibers; and 5 = randomly oriented fibers.

### 4.8. Biomechanics

After extraction, the Achilles tendon specimens were immediately frozen at −20 °C in gauze soaked in NaCl solution. Before tensile testing at room temperature, the tendons were thawed overnight at 4 °C and slowly warmed to RT. All tendons were harvested from the hind legs, including the muscle and the calcaneus. On the muscle side, the samples were mounted in serrated clamps after being wrapped in two pieces of cloth to reduce slippage, and on the bone side, a device fixing the calcaneus in a rectangular position to the tendon was used. All samples were tested in uniaxial tension to failure at a 1 mm min^−1^ speed on a universal material testing machine (Zwick 1456, 1 kN load-cell, TestXpert 10, Germany) with preconditioning (10 cycles to 10 N). The samples were sprayed with phosphate-buffered saline during measurement in order to prevent drying. The load until failure (N) was determined as the maximum load measured.

The cross-sectional area (CSA) was determined 2 cm above the calcaneus by a custom-designed linear laser scanner (n = 3 per specimen) before the tensile testing. The load until failure (N) was divided by the thus determined CSA at the repair site (mm^2^), resulting in the failure stress at the repair site (MPa). The stiffness was assessed in the load-elongation curve and the elastic modulus (E-Modulus; MPa) was determined from the stress–strain curves.

### 4.9. Statistical Evaluation

Data in the text are represented as means ± standard deviations (SDs). Data were analyzed and visualized with Microsoft Excel (version 16.77, USA) and the GraphPad Prism software (version 10.0.2, USA). The Shapiro–Wilk test was applied to analyze the normality of the data distribution. Comparisons between groups were performed with the Kruskal–Wallis test when data were not normally distributed and/or variance homogeneity was not given. In case of normal distribution, statistical analysis was carried out with ordinary one-way ANOVA using Tukey’s multiple comparison test or unpaired *t*-test. A *p*-value ≤ 0.05 was considered statistically significant. Contingency tables were evaluated by calculation of the contingency coefficient, and *p*-values ≤ 0.05 for the pairwise comparison were considered significant.

## 5. Conclusions

In this study, we co-cultured rabbit ASCs and rabbit tenocytes in a 3:1 ratio and collected their secretome. After concentrating it a hundred times, it was incorporated in DP polymer fibers by emulsion electrospinning to yield secretome tubes that were characterized by their fiber thickness, pore size, static and dynamic WCA, and FTIR. Next, the bilayer secretome tubes were implanted in the rabbit Achilles tendon full transection model, where, 3 weeks post-operation, the adhesion formation was significantly reduced. The morphology of secretome-treated tendons included a significantly lower CSA than tendons treated with a pure DP tube. Furthermore, we determined a peak load and stiffness that was not significantly different from native tendons, which was not the case for the DP group, where these two parameters were significantly lower.

We concluded that a combination of a secretome-releasing tube and an initial injection of concentrated secretome harvested from an ASC/tenocyte co-culture has promising effects on tendon healing, addressing the most prominent problems during tendon healing, namely adhesion formation and poor biomechanics. Thus, a cell-free approach could be used to support tendon healing in clinics in the future.

## Data Availability

Data are contained within the article.

## Supplementary Materials

The following supporting information can be downloaded at https://www.mdpi.com/article/10.3390/ijms26125457/s1.
