# Supplementary material for: Electrospun DegraPol Tube Delivering Stem Cell/Tenocyte Co-Culture-Derived Secretome to Transected Rabbit Achilles Tendon—In Vitro and In Vivo Evaluation
Source: Int J Mol Sci. 2025 Jun 6;26(12):5457. doi: 10.3390/ijms26125457 (PMC12192585; doi:10.3390/ijms26125457)
Supplement: Supplementary file 1 [file ijms-26-05457-s001.zip › ijms-3634489-supplementary.pdf]

Supporting Information

Electrospun DegraPol tube delivering stem cell/tenocyte co-culture-derived secretome to fully transected rabbit Achilles tendon – an in vitro and in vivo evaluation

Julia Rieber [1], Iris Miescher [1], Petra Wolint [1], Gabriella Meier Bürgisser [1], Jeroen Grigioni [2], Jess G. Snedeker [2,3], Viola Vogel [4], Pietro Giovanoli [1], Maurizio Calcagni [1] and Johanna Buschmann [1*]

A

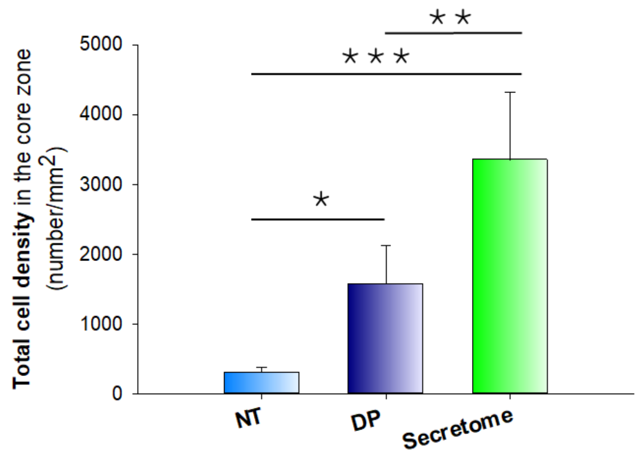

B

Score	1.0	1.5	2.0	2.5	3.0	3.5	4.0	4.5	5.0	Total
DP	1	0	1	2	5	2	4	0	0	**15**
NT	23	7	0	0	0	0	0	0	0	**30**
Secretome	1	1	7	5	9	10	3	0	0	**36**
Total	**25**	**8**	**8**	**7**	**14**	**12**	**7**	**0**	**0**	**81**

Figure S1. Total cell density assessed in H&E stained histological sections in the core tissue of the Achilles tendon (**A**) for the groups NT = not treated, DP = pure DegraPol® tube, and Secretome = Achilles tendons that received a secretome releasing tube and an injection of 50 µL of secretome. A Kruskal-Wallis test was used to compare the means because variance homogeneity of data was not given (despite a normal distribution of data) with p values < 0.05 (*), < 0.01 (**) and < 0.001 (***). Collagen fiber orientation semi-quantitatively assessed with scores 1-5 (and half steps) for DP, NT and Secretome group, respectively (**B**). While contingency coefficient analysis revealed a significant difference between DP-NT and Secretome-NT in pairwise comparison, the comparison of DP and Secretome was not significantly different.
